# *Cornus macrophylla*, the Antibacterial Activity of Organic Leaf Extracts and the Characterization of the More Lipophilic Components by GC/MS

**DOI:** 10.3390/molecules25102395

**Published:** 2020-05-21

**Authors:** Muhammad Akbar, Usman Ali, Tayyaba Khalil, Muhammad Sajjad Iqbal, Awais Amin, Rehan Naeem, Abdul Nazir, Hafiz Muhammad Waqas, Zohaib Aslam, Faisal Iqbal Jafri, Nazir Aslam, Safeer Akbar Chohan

**Affiliations:** 1 Department of Botany, University of Gujrat, Gujrat 50700, Pakistan; usmanbutt874@gmail.com (U.A.); tayyaba.khalil@uog.edu.pk (T.K.); drsajjad.iqbal@uog.edu.pk (M.S.I.); awaisamin043@gmail.com (A.A.); vk6296717@gmail.com (H.M.W.); mirzathecupid74@gmail.com (Z.A.); faisal.bhs@gmail.com (F.I.J.); nazirmbdin@gmail.com (N.A.); meriuog@gmail.com (S.A.C.); 2 Department of Biotechnology and Genetic Engineering, Kohat University of Science and Technology, Kohat 26000, Khyber Pakhtunkhwa, Pakistan; gulerehan@gmail.com; 3 Department of Environmental Sciences, COMSATS University Islamabad, Abbottabad Campus, Tobe Camp, Abbottabad 22060, Khyber Pakhtunkhwa, Pakistan; abdulnazeer@cuiatd.edu.pk

**Keywords:** α-amyrin, antibacterial activity, *Cornus macrophylla*, GC/MS

## Abstract

In the present study, the antibacterial activity of *Cornus macrophylla* was examined. Organic solvent extracts of leaves were prepared using methanol, *n-*hexane, chloroform, and ethyl acetate. Antibacterial activity was examined by using a 100 mg/mL extract concentration. Penicillin was kept as a positive control while dimethyl sulfoxide was taken as a negative control. Methanolic extract exhibited a 21.5, 36.3, 25.3, and 23.7 mm inhibition zone diameter (IZD); *n*-hexane showed a 33, 40, 32.8, and 28.7 mm IZD; chloroform showed a 18.8, 29, 22.3, and 21.6 mm IZD; and ethyl acetate showed a 23.5, 30.2, 30, and 22.3 mm IZD against *Erwinia carotovora, Pseudomonas syringae, Ralstonia solanacearum*, and *Xanthomonas axonopodis,* respectively. The *n*-hexane extract revealed high antibacterial activity against all bacterial species as compared with methanolic, chloroform, and ethyl acetate extract. Gas Chromatography Mass Spectrometry (GC/MS) analysis of *n*-hexane extract depicted the presence of 55 compounds. Out of these compounds, one compound, identified as α-amyrin (Mol. wt = 426), exhibited the maximum peak area (32.64%), followed by A’-Neogammacer-22(29)-en-3-ol, acetate, (3.beta.,21.beta.)- (Mol. wt = 468) and β-amyrin (Mol. wt = 426) having peak areas of 25.97 and 6.77%, respectively. It was concluded that the antibacterial activity observed during the present investigation may be due to these compounds.

## 1. Introduction

Plants are a valuable source of bioactive compounds due to the production of secondary metabolites. Secondary metabolites of plants show antimicrobial activity against a number of pathogens [1,2]. The extracts of plants are also used for treatment of serious diseases [3]. For the maintenance of quality and quantity of food, there is a need to control plant diseases caused by various pathogens. At present, the most reliable method for controlling bacterial pathogens is the use of synthetic/chemical pesticides. Although pesticides are helpful to crops, they have negative impacts on biodiversity, pollute the environment [4,5], and cause health problems [6,7]. Also, bactericidal application kills microbes that help plants defend against pathogens [8]. Moreover, numerous pathogens have developed resistance against numerous synthetic/commercial antibacterial compounds. These negative effects of synthetic chemicals have increased the interest of researchers in exploring natural biodegradable antimicrobials that could be effective alternatives to synthetic chemicals [9,10,11].

Approximately 20,000 plants around the world have medicinal activities; these plants have different bioactive compounds [12]. These bioactive compounds have antimicrobial activities and control the attack of plant pathogens on crops [13]. Plants have been studied widely due to their potent bioactive compounds and recent studies showed that different parts of plant extracts have antimicrobial activities due to the presence of these bioactive compounds [14,15]. *Cornus macrophylla* belongs to the family Cornaceae, which consists of 55 species. Three species of this family, viz., *C. macrophylla, Cornus oblonga,* and *Cornus capitata*, are present in Pakistan [16]. Research carried out during the recent past has shown that plants in genus *Cornus* are a source of beneficial bioactive compounds. *C. macrophylla* is a medicinal plant. Its bark can be used orally in powder form or in black tea to treat backache, jaundice, and stomach ulcers [17]. *C. macrophylla* also exhibited a strong inhibition of aldose reductase, and it may be a potential candidate for the treatment of diabetic retinopathy [18]. In some previous studies, various biological activities such as the antifungal [19], antibacterial [20], and antioxidant [21] activities of numerous compounds and/or complexes isolated from *Cornus* spp. were investigated. The use of plants that produce antimicrobial compounds is an alternative way to control bacterial attack [8]. By using antimicrobial plants for the protection of crops against pathogens, we can decrease the development of resistance in pathogens caused by synthetic chemical compounds [22]. Aqueous extract of *Rhus coriaria* showed antibacterial activity and Gas Chromatography Mass Spectrometry (GC/MS) of its aqueous extract showed the presence of 39 compounds. Of these 39 compounds, 2,5-Furandione was documented as having the best antibacterial activity [23]. There are few studies on the isolation of antimicrobial compounds from the genus *Cornus.* Phytochemical screening of *C. macrophylla* as a whole plant revealed the presence of alkaloids, steroids, terpenoids, flavonoids, reducing sugars, and tannins. The ethyl acetate, methanolic extracts, and crude extracts showed the presence of alkaloids, terpenoids, tannins, and reducing sugars, while *n*-hexane, chloroform, and ethyl acetate fractions revealed the existence of terpenoids, steroids, flavonoids, tannins, and reducing sugars. The crude and methanolic extracts exhibited antibacterial activity [a 14 mm inhibition zone diameter (IZD)] at a concentration of 32 mg/mL [24]. The ethyl acetate extract of *C. macrophylla* leaves exhibited antibacterial activity against *Pseudomonas aeruginosa*, a human pathogen. A compound named as kaempferol 3-*O*-α-L-rhamnopyranoside (afzelin) was isolated from *C. macrophylla* leaves. The minimum inhibitory concentration (MIC) of afzelin was found to be 31 µg/mL against *P. aeruginosa*; however, the antibacterial activity shown by afzelin was less than gentamycin, the reference compound [18]. Compounds isolated from the methanolic extract of the fruit of *Cornus mas* were β-hydroxychalcone, 4-acetoxy-5,20,40,60, β-pentahydroxy-3-methoxychalcone,7,30-dihydroxy-5,40-dimethoxyflavanone, myricetin, quercetin-3-*O*-rutinoside, gallic acid, ursolic acid, and d-glucose. These compounds exhibited antibacterial activity against *Staphylococcus aureus* and *Pseudomonas aeruginosa* [25].

Although there are a few investigations on the isolation of antibacterial compounds against animal pathogens from *C. macrophylla* around the world, reports on the characterization of antibacterial compounds against plant pathogens are missing. Therefore, the present study would be a useful addition to research work. In the present study, phytochemicals were characterized/identified from *C. macrophylla* and tested as having antibacterial activities against some plant pathogenic bacterial species, viz., *Erwinia carotovora, Ralstonia solanacearum, Xanthomonas axonopodis,* and *Pseudomonas syringae*. Infections due to these bacteria cause serious threats to food security [26].

There are also numerous diseases in plants caused by plant pathogenic bacteria. Plant pathogenic bacteria have a serious effect on crops and reduce the yield of crops [27]. A bacterial disease caused by *P. syringae* is bacterial blight of wheat, which reduces the yield of wheat every year [28]. Bacterial wilt disease in Solanaceae is caused by *R. solanacearum* [29], especially in tomato [30]. Similarly, *Xylella fastidiosa* causes disease in citrus plants [31]. In Rosaceae, fire blight is a disease caused by *Erwinia amylovora* [32]. *E. carotovora* is responsible for Cassava bacterial stem rot [33]. Similarly, *X. axonopodis* is responsible for cankers on *Citrus maxima* [34]. Additionally, *Xanthomonas campestris* pv. *Mangiferae indicae* is responsible for mango bacterial canker disease [35]. All of these plant pathogens have a broad host range and cause a number of diseases in many plants. Therefore, the present study was designed to assess the *in vitro* antibacterial activity of bioactive compounds of *C. macrophylla* separated through methanol, *n-*hexane, chloroform, and ethyl acetate. The metabolites in the most active organic fraction from *C. macrophylla* were identified with the help of GC/MS and have not been reported in earlier investigations. This study could help to further extend our knowledge of bioactive molecules that can be harnessed as natural eco-friendly antibacterial compounds.

## 2. Results and Discussion

**Figure 1:**
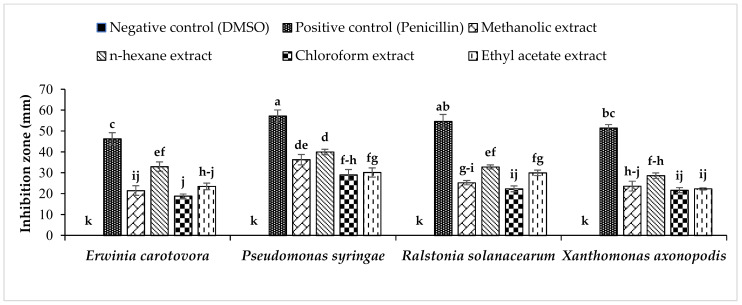
Effect of different organic solvent extracts of *Cornus macrophylla* leaves on the growth of *Erwinia carotovora*, *Pseudomonas syringae*, *Ralstonia solanacearum,* and *Xanthomonas axonopodis*. Vertical bars show the standard error of means of three replicates. Values with different letters show a significant difference (*p* ≤ 0.05) as determined by ANOVA followed by Fisher’s least significant difference (LSD) Test using Minitab statistical software (Minitab 19).

Figure 1 shows the antibacterial activity of *C. macrophylla* leaf extracts against *E. carotovora*, *P. syringae*, *R. solanacearum*, and *X. axonopodis*. In all of these experiments, DMSO kept as a negative control did not show any antibacterial activity while penicillin used as a positive control exhibited the maximum antibacterial activity in terms of IZD.

### 2.1. Antibacterial Activity of Methanolic, n-hexane, Chloroform, and Ethyl Acetate Extract of C. macrophylla Leaves on E. carotovora

Methanolic extract significantly exhibited a 21.5 mm IZD against *E. carotovora* while penicillin showed a 46.3 mm IZD. The *n*-hexane extract revealed the maximum antibacterial activity as compared with methanolic, chloroform, and ethyl acetate extract. The *n*-hexane extract showed a 33 mm IZD against *E. carotovora*, whereas extract of chloroform showed an 18.8 mm IZD, which was less than all other extracts. The ethyl acetate extract also showed substantial results, forming an IZD of 23.5 mm. In the case of organic solvent fractions, a maximum 33 mm IZD was recorded. In the case of the *n*-hexane extract of *C. macrophylla* leaves, this IZD was less than the penicillin used as a positive control (Figure 1). These results showed similarities to the findings of [36] in which researchers investigated the effect of *Urospermum picroides* against *E. carotovora* and recorded an inhibition zone of 7–8 mm. Inhibition caused by the organic solvent extract of *C. macrophylla* leaves on *E. carotovora* was greater than that caused by *U. picroides*. In a previous study, an ethyl acetate fraction of *Amaranthus viridis* leaf exhibited a 19 mm IZD against *E. carotovora* [37]. This higher efficacy can be attributed to a greater amount of antibacterial substances present in the leaves of *C. macrophylla*.

### 2.2. Antibacterial Activity of Methanolic, n-hexane, Chloroform, and Ethyl Acetate Extract of C. macrophylla Leaves on P. syringae

Methanolic extract exhibited a 36.3 mm IZD against *P. syringae* whereas penicillin showed a 57.2 mm IZD. The *n*-hexane extract revealed the best antibacterial activity as compared with chloroform and ethyl acetate extract, exhibiting a 40 mm IZD against *P. syringae*. Extract of chloroform showed a 29 mm IZD, which was less than all other extracts. Ethyl acetate extract also showed significant results with an IZD of 30.2 mm (Figure 1). These results showed similarities to the findings of [38] in *Polygonum cuspidatum* roots against *P. syringae* and exhibited 100% inhibition after 24 hours at a 105.11 µg/mL concentration. In another study, an ethyl acetate fraction of *A. viridis* leaf caused a 21 mm IZD against *P. syringae* [37].

### 2.3. Antibacterial Activity of Methanolic, n-hexane, Chloroform, and Ethyl Acetate Extract of C. macrophylla Leaves on R. solanacearum

The antibacterial activity of methanolic extract of *C. macrophylla* leaves is shown in Figure 1. Methanolic extract exhibited a 25.3 mm IZD against *R. solanacearum* whereas the corresponding value for penicillin was 54.7 mm. The *n*-hexane extract revealed more potent antibacterial activity than chloroform and ethyl acetate extracts. The *n*-hexane extract showed a 32.8 mm IZD against *R. solanacearum*. The extract of chloroform showed a 22.3 mm IZD, which was less than all other extracts. The ethyl acetate extract also showed significant results (a 30 mm IZD). In this experiment, *n*-hexane showed maximum antibacterial activity. In an earlier investigation, the methanolic extract of *R. coriaria* exhibited an 18 mm zone of inhibition against *R. solanacearum* [21]. Ethanolic extract of *Ipomoea staphylina* has antibacterial activity against *Xanthomonas campestris*, *P. syringae*, *Klebsiella pneumonia*, *Escherichia coli*, *Salmonella typhi*, *P. aeruginosa* and *S. aureus*. GC/MS analysis of the ethanolic extract revealed the presence of alkaloids, saponins, flavonoids, steroids, glycosides, phenols, and sterols [39].

### 2.4. Antibacterial Activity of Methanolic, n-hexane, Chloroform, and Ethyl Acetate Extract of C. macrophylla Leaves on X. axonopodis

Figure 1 shows the data on the antibacterial activity of *C. macrophylla* extracts against *X. axonopodis*. Methanolic extract exhibited a 23.7 mm IZD against *X. axonopodis* whereas penicillin showed a 51.5 mm IZD. The *n*-hexane extract revealed substantial antibacterial activity as compared with methanol, chloroform, and ethyl acetate extract. The *n*-hexane extract showed a 28.7 mm IZD against *X. axonopodis*. On the other hand, the extract of chloroform showed a minimum (21.7 mm IZD) bactericidal activity. The ethyl acetate extract also showed significant results (a 22.3 mm IZD). A maximum IZD of 28.7 mm was recorded in the case of *n*-hexane extract, which was less than that of penicillin. These results are in agreement with the findings of [40] where *Amaranthus tricolor* showed 24%–62% antibacterial activity against *X. axonopodis*.

### 2.5. Gas Chromatography Mass Spectrometry (GC/MS) Analysis

**Figure 2:**
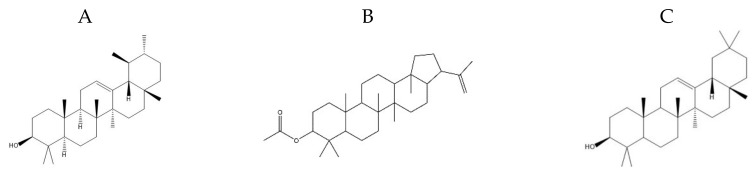
(**A**–**C**). Chemical structures of (**A**) α-amyrin, (**B**) A’-Neogammacer-22(29)-en-3-ol, acetate, (3.beta.,21.beta.)-, and (**C**) β-amyrin.

In total, 55 compounds were identified in the *n-*hexane fraction of *C. macrophylla*. The retention time (RT), peak areas of component (%), molecular weight, and their molecular formulas are presented in Table 1. Of these compounds, only three compounds revealed >5% peak areas, viz., α-amyrin; A’-Neogammacer-22(29)-en-3-ol, acetate, (3.beta.,21.beta.)-; and β-amyrin (Figure 2A–C). The antibacterial activity of α-amyrin and β-amyrin was also reported against *S. aureus*, *Bacillus subtilis*, *Enterococcus faecium* and *Staphylococcus saprophyticus* [41,42]. Both α- and β-amyrin triterpenes have also been isolated from *Dorstenia arifolia* and documented as having antimicrobial activities [43]. The compounds α-, β-amyrin, and α-amyrin phenylacetate reduced the bacterial viability to less than 20% [44]. *S. aureus* (MRSA) is an important human pathogen that has become resistant to antibiotics. The compound α-amyrin has been reported to exhibit antimicrobial activities against *S. aureus*. The compound α-amyrin regulates multiple desirable targets in cell division, the two-component system, ABC transporters, fatty acid biosynthesis, peptidoglycan biosynthesis, aminoacyl-tRNA synthetase, and ribosome and b-lactam resistance pathways [45], resulting in the destabilization of the bacterial cell membrane, a halt in protein synthesis, and inhibition of cell growth that eventually lead to cell death [46]. Furthermore, it causes disorganizing effects on cardiolipin-rich domains present in the membrane of *E. coli* [47]. The α-amyrin identified from *Pyrus bretschneideri* Rehd. also exhibited antibacterial activity [48]. Moreover, α- and β-amyrin esters are also documented as antibacterial compounds [49]. In another investigation, β-amyrin isolated from leaves of *Siraitia grosvenorii* showed antibacterial activity against *Streptococcus mutans, Actinobacillus actinomycetemcomitans,* and *Fusobacterium nucleatum* with minimum inhibitory concentrations of 48.80, >100, and 48.80 μg mL^−1^, respectively [50]. On the other hand, there are no previous reports that describe the antibacterial activity of A’-Neogammacer-22(29)-en-3-ol, acetate. In the present study, a higher level antibacterial activity of the *n*-hexane extract of *C. macrophylla* leaves was recorded as compared with chloroform and ethyl acetate extracts; *n*-hexane is a non-polar solvent and has a greater ability to extract more lipophilic compounds like α-amyrin, as compared with chloroform and ethyl acetate. Since GC/MS of *n*-hexane extract of *C. macrophylla* leaves from Pakistan has shown the presence of α-amyrin having the highest peak area, more studies are required to isolate and characterize its bioactive constituents.

## 3. Materials and Methods

### 3.1. Collection and Identification of Plant Material

Fresh leaves of *C. macrophylla* were collected from the Bara Gali summer campus, University of Peshawar, Khyber Pakhtunkhwa (KPK), Galyat, Pakistan. The voucher specimen (UOG-000585) was deposited in the herbarium of the Department of Botany, University of Gujrat, Gujrat, Pakistan.

### 3.2. Preparation of C. macrophylla Leaves Extracts

After collection, leaves of *C. macrophylla* were sun dried for 1 week and dried leaves (1 kg) were ground with the help of a pestle and mortar to make a fine powder. The powder (400 g) was soaked in 1-L of methanol in a glass jar and incubated for 1 week at room temperature (25 °C) and frequently stirred with a glass rod. The filtration of the extract was performed by using four layered muslin cloth followed by a final filtration with Whatman filter paper No. 1. The filtrate was evaporated at 45 °C by using a rotary evaporator (Model: Laborata 4000/Gl, Heidolph, Schwabach, Germany). Extra methanol from this extract was evaporated under currents of clean air at room temperature to yield a viscous fluid termed as methanolic extract. This methanolic extract was reconstituted in double-distilled water (200 mL) and fractionated with three organic solvents, viz., *n-*hexane, chloroform, and ethyl acetate, first with 200 mL of *n-*hexane in a 500 mL separating funnel. This setup was left overnight until the *n-*hexane formed a layer in the upper portion of the separating funnel, which was then separated into a glass beaker. The process was repeated thrice by adding fresh solvent into the aqueous solution. A similar process was used for the extraction with chloroform and ethyl acetate. The organic solvent extracts thus obtained were evaporated by using a rotary evaporator, as discussed earlier, and stored at 4 °C until further use.

### 3.3. Culturing of Target Plant Pathogenic Bacterial Species

Plant pathogenic bacterial cultures were obtained from the Culture Bank of Pakistan, University of the Punjab, Lahore, Pakistan. The bacterial cultures with their accession numbers were *E. carotovora* (FCBP-PB-0421)*, P. syringae* (FCBP-PB-0405), *R. solanacearum* (FCBP-PB-0407), and *X. axonopodis* (FCBP-PB-001). These cultures were sub-cultured on a Lysogeny broth (LB) medium in 9 cm diameter glass petri plates until colonies became visible and stored in a refrigerator at 4 °C for further use.

### 3.4. Preparation of Control and Stock Solutions, Culture Medium, and Antibacterial Assays

For antibacterial assays, a disk diffusion method was adopted according to the procedure described in our previous publication, with slight modifications [51]. For the preparation of the negative control solution, 166 µL of DMSO was mixed with 333 µL of autoclaved distilled water to make a final volume of 500 µL and for the preparation of the positive control solution, 50 mg of penicillin was dissolved in 166 µL DMSO and 333 µL of autoclaved distilled water was added to make a volume of 500 µL. Stock solutions of organic solvent extracts were prepared in a way similar to the preparation of the positive control solution. 50 mg of leaves extract in each organic solvent viz. methanol, *n*-hexane, chloroform and ethyl acetate were dissolved into 166 µL of DMSO and then added 333 µL of autoclaved distilled water to make volume up to 500 µL. In this way, the positive control, penicillin, and all organic solvent extracts of *C. macrophylla* leaves were tested for their antibacterial efficacy at a 100 mg mL^−1^ concentration. The LB medium was used for inoculation of bacterial species. For the preparation of the LB medium, 1000 mL of distilled water was added into the conical flask, then 5 g of yeast extract, 10 g of tryptone, 10 g of NaCl, and 15 g of agar powder were added and mixed well to dissolve all the nutrients. Afterwards, the flask opening was covered with aluminum foil and sterilized in autoclave for 20 min at 121 °C. After preparing the LB agar plates, bacterial inocula @ 1 × 10^5^ cfu/mL were spread evenly onto these plates and, after spreading, filter paper discs (6 mm) were placed on these plates. Leaf extract (25 µL) for each solvent (methanol, *n*-hexane, chloroform, and ethyl acetate) was added onto these filter paper discs contained in Petriplates and incubated at 37 °C. Antibacterial activity was measured after 72 h in terms of inhibition zone diameter (IZD) with the help of a measuring scale [37]. All chemicals used were of Merck KGaA, Darmstadt, Germany.

### 3.5. Gas Chromatography Mass Spectrometry (GC/MS)

Constituents of *n-*hexane extract of *C. macrophylla* leaves showing higher bioactivity were analyzed by using GC/MS on a Clarus 500 Mass Spectrometer (PerkinElmer, Waltham, Massachusetts, USA) whose detectable mass range was set at 35–500 *m/z*. The ion source and interface temperatures were 200 °C and 250 °C, respectively. The start and end times were 2.50 min and 47.14 min, respectively. The column oven temp. was 40 °C whereas the injection temp. was 25 °C. Injection mode was split and flow control mode was set at a pressure of 100 kPa. Total flow was 13.9 mL/min while column flow was 1.78 mL/min with a linear velocity of 48.1 cm/sec. Purge flow was kept at 3.0 mL/min and a split ratio of 5.1. The oven temperature was programmed first at 40 °C for 5 min with an increase of 5 °C min^−1^ to 80 °C, then 5 °C min^-1^ to 300 °C for 5 min. The mass spectral library consulted for GC/MS analysis for the identification of components in our study was NIST14.lib. This part of the research was conducted at the Thermal Energy Research Lab., National University of Sciences and Technology, Islamabad, Pakistan.

### 3.6. Statistical Design and Analysis

The experiment was performed by adopting Completely Randomized Design (CRD). For statistical analysis, ANOVA was done followed by Fisher’s least significant difference (LSD) Test using Minitab Statistical Software (Minitab 19, State College, Pennsylvania, USA).

## 4. Conclusions

The present study revealed the antibacterial efficacy of *C. macrophylla* leaf extracts. GC/MS analysis of *n*-hexane extract depicted the presence of α-amyrin having the highest peak area % age. It may be concluded that this compound, having the highest peak area % age, was responsible for the antibacterial activity recorded in the present study. The structure of this compound can be utilized further to develop eco-friendly bactericides in the future.

## Figures and Tables

**Table 1:** Compounds Identified in Gas Chromatography Mass Spectrometry Analysis.

Sr. #	Retention Time (min.)	Name of Compound	Molecular Formula	Molecular Weight	Peak Area%	Class of Compound
1	4.255	3-Hexanone	C_6_H_12_O	100	0.01	Di alkyl Ketone
2	4.389	2-Hexanone	C_6_H_12_O	100	0.01	Ketone
3	22.776	2,4-Di-tert-butylphenol	C_14_H_22_O	206	0.01	Phenol
4	24.436	Nonadecane	C_19_H_40_	268	0.02	Aliphatic Alkane
5	26.146	Heptadecane	C_17_H_36_	240	0.02	Aliphatic Alkane
6	27.771	Heneicosane	C_21_H_44_	296	0.03	Aliphatic Alkane
7	28.339	Neophytadiene	C_20_H_38_	278	0.56	Terpene
8	28.419	2-Pentadecanone, 6,10,14-trimethyl-	C_18_H_36_O	268	0.03	Terpenoid
9	29.682	Hexadecanoic acid, methyl ester	C_17_H_34_O_2_	270	0.11	Saturated Fatty acid
10	30.678	Hexadecanoic acid, ethyl ester	C_18_H_36_O_2_	284	0.04	Saturated Fatty acid
11	32.098	9,12-Octadecadienoic acid (*Z,Z*)-, methyl ester	C_19_H_34_O_2_	294	0.06	Unsaturated fatty acid
12	32.183	9,12,15-Octadecatrienoic acid, methyl ester, (*Z,Z,Z*)-	C_19_H_32_O_2_	292	0.26	Unsaturated fatty acid
13	32.556	Methyl stearate	C_19_H_38_O_2_	298	0.02	Fatty acid
14	32.824	9,12-Octadecadienoic acid (*Z,Z*)-	C_18_H_32_O_2_	280	1.09	Unsaturated fatty acid
15	33.005	Linoleic acid ethyl ester	C_20_H_36_O_2_	308	0.17	Unsaturated fatty acid
16	33.092	9,12,15-Octadecatrienoic acid, ethyl ester, (*Z,Z,Z*)-	C_20_H_34_O_2_	306	0.31	Fatty acid
17	33.175	2,2-Dimethyl-6-methylene-1-[3,5-dihydroxy-1-pentenyl]cyclohexan-	C_14_H_24_O_4_	256	0.08	Phenolic
18	33.708	Phytol, acetate	C_22_H_42_O_2_	338	0.06	Terpene
19	34.360	Ergost-25-ene-3,6-dione, 5,12-dihydroxy-, (5.αalpha.,12.beta.)-	C_28_H_44_O_4_	444	0.15	Ester
20	34.824	Eicosane	C_20_H_42_	282	0.09	Aliphatic Alkane
21	35.457	2,5-Bis(1,1-dimethylbutyl)-4-methoxyphenol	C_19_H_32_O_2_	292	0.28	Phenolic
22	35.546	Urs-12-ene	C_30_H_50_	410	0.17	Tri-Terpenoid
23	35.721	4,4,6a,6b,8a,11,11,14b-Octamethyl-1,4,4a,5,6,6a,6b,7,8,8a,9,10,11,12,12a,14,14a,14b-octadecahydro-2H-picen-3-one	C_30_H_48_O	424	0.32	Tri-Terpenoid
24	36.080	2-Methyltetracosane	C_25_H_52_	352	0.03	Tri-Terpenoid
25	37.098	Spiro[androst-5-ene-17,1’-cyclobutan]-2’-one, 3-hydroxy-, (3.	C_22_H_32_O_2_	328	0.02	Steroid
26	37.274	Tetracosane	C_24_H_50_	338	0.04	Alkane
27	37.810	22,23-Dibromostigmasterol acetate	C_31_H_50_Br_2_O_2_	612	0.50	Steroid Ester
28	37.880	Urs-12-ene-3.beta.,11.beta.-diol, diacetate	C_34_H_54_O_4_	526	0.69	Tri-Terpenoid
29	37.955	13,27-Cyclours-11-en-3-ol, acetate	C_32_H_50_O_2_	466	0.89	Ester
30	38.280	Ether, dodecyl isopropyl	C_15_H_32_O	228	0.01	Ether
31	38.350	Undec-10-ynoic acid, decyl ester	C_21_H_38_O_2_	322	0.02	Ester
32	38.416	Dotriacontane, 1-iodo-	C_32_H_65_I	576	0.01	Alkane
33	38.552	9,19-Cyclolanost-24-ene-3,26-diol, diacetate	C_34_H_54_O_4_	526	0.17	Diester
34	38.810	3,7,11,15-Tetramethyl-2-hexadecen-1-ol	C_20_H_40_O	296	0.13	Alkane
35	38.884	13,14-Epoxyursan-3-ol, acetate	C_31_H_50_O_3_	470	0.12	Ester
36	39.204	Olean-12-en-3-ol, acetate, (3.beta.)-	C_32_H_52_O_2_	468	3.10	Ester
**37**	**39.307**	**β** **-amyrin**	**C_30_H_50_O**	**426**	**6.77**	**Ester**
38	39.407	β.-Amyrone	C_30_H_48_O	424	1.21	Ester
39	39.848	Lup-20(29)-en-3-one	C_30_H_48_O	424	1.17	Tri-Terpenoid
40	40.012	1,4-Benzenedicarboxylic acid, bis(2-ethylhexyl) ester	C_24_H_38_O_4_	390	0.11	Benzene Carboxylic Acid
41	40.380	Squalene	C_30_H_50_	410	0.93	Tri-Terpenoid
**42**	**40.923**	**α** **-amyrin**	**C_30_H_50_O**	**426**	**32.64**	**Tri-Terpenoid**
43	41.621	Tetracontane	C_40_H_82_	562	2.57	Alkane
44	42.133	Thunbergol	C_20_H_34_O	290	2.65	Steroid
45	42.426	Cholest-5-en-3-ol (3.beta.)-, carbonochloridate	C_28_H_45_ClO_2_	448	2.63	Steroid
46	42.750	Octacosyl acetate	C_30_H_60_O_2_	452	2.18	Fatty Alcohol
**47**	**43.300**	**A’-Neogammacer-22(29)-en-3-ol, acetate, (3.beta.,21.beta.)-**	**C_32_H_52_O_2_**	**468**	**25.97**	**Ester**
48	43.735	Hexatriacontane	C_36_H_74_	506	2.12	Aliphatic Alkane
49	43.909	Stigmast-5-en-3-ol, oleate	C_47_H_82_O_2_	678	2.15	Ester
50	44.306	Acetyl betulinaldehyde	C_32_H_50_O_3_	482	3.68	Tri-Terpenoid
51	44.600	Silane, chlorodiethyl(dodec-9-ynyloxy)-	C_16_H_31_ClOSi	302	0.79	Alkane
52	44.990	Lanosta-8,24-dien-3-ol, acetate, (3.beta.)-	C_32_H_52_O_2_	468	1.69	Ester
53	46.150	Pentadecanophenone	C_21_H_34_O	302	0.27	Ketone
54	46.404	Acetic acid, 4,4,6a,6b,8a,11,12,14b-octamethyl-14-oxo-1,2,3,4,4a,	C_32_H_50_O_3_	482	0.54	Carboxylic acid
55	46.783	Ergosta-5,22-dien-3-ol, (3.beta.,22E)-	C_28_H_46_O	398	0.30	Cholesterol
					Total 100%	

Note: Compounds highlighted in bold were detected as having higher peak area percentages (>5%).
